# “The Sheep Did It Again”: Replication of Animal-Assisted Treatment in Psychiatric Inpatients with Substance Use Disorder and Borderline Personality Disorder in a Randomized Controlled Trial

**DOI:** 10.3390/healthcare13212808

**Published:** 2025-11-05

**Authors:** Petra Schmid, Carmen Nauss, Claudia Jauch-Ederer, Petra Prinz, Anna Lena Kordeuter, Stefan Tschöke, Carmen Uhlmann

**Affiliations:** 1 Klinik für Psychiatrie und Psychotherapie I (Weissenau), Universität Ulm, 89081 Ulm, Germany; carmen.nauss@zfp-zentrum.de (C.N.); claudia.jauch-ederer@zfp-zentrum.de (C.J.-E.); annalena.kordeuter@zfp-zentrum.de (A.L.K.); stefan.tschoeke@zfp-zentrum.de (S.T.); carmen.uhlmann@zfp-zentrum.de (C.U.); 2 ZfP Südwürttemberg, Versorgungsforschung, 88214 Ravensburg, Germany; 3 Prinzenhof-Leutkirch, 88299 Leutkirch, Germany; prinzenhof-leutkirch@web.de

**Keywords:** animal-assisted services, animal-assisted intervention, emotion regulation, human–animal interaction, mindfulness, self-efficacy expectancy

## Abstract

Background: In an initial pilot study, we investigated an animal-assisted treatment (AAT) procedure with sheep as an adjunct to treatment as usual (TAU+AAT) in psychiatric inpatients with substance use disorder (SUD). Over time, this TAU+AAT intervention significantly reduced negative emotions and improved positive emotions, mindfulness, and self-efficacy expectancy compared to TAU. In the current study, we aimed to replicate these results and extend the investigation to another group of inpatients with difficulties in emotion regulation, namely borderline personality disorder (BPD). Methods: A single-session AAT procedure with sheep in a group setting as an adjunct to treatment as usual (TAU+AAT) was examined in an RCT compared to TAU. A total of 29 psychiatric inpatients with SUD and 31 with BPD were examined (PRE vs. POST) using questionnaires on variables that included positive and negative emotions, mindfulness, and self-efficacy expectations. Results: In the SUD sample, significant effects between PRE and POST, with large effect sizes in all four outcomes, emerged for the TAU+AAT group, in contrast to TAU. In the BPD sample, similar time (PRE vs. POST) and group (TAU+AAT vs. TAU) effects were achieved for all outcomes. Conclusions: Repeatedly, this TAU+AAT intervention, which involved a single session with sheep, improved in all outcomes. Sheep seem to be suitable for AAT with a focus on mindfulness.

## 1. Introduction

Emotions are an essential part of human existence, but sometimes they are experienced inappropriately in terms of their intensity degree or context [1]. Emotion regulation encompasses the ability to deal with and modulate affective experiences [2]. Deficits in emotion regulation are associated with the development and maintenance of mental disorders [3], such as substance use disorder (SUD) or borderline personality disorder (BPD). In SUD, cognitive–behavioral models of disorder assume a higher degree of negative emotionality and impairments in emotion regulation, which lead to impulsive actions [4,5]. BPD is characterized by the occurrence of a high degree of overwhelming, unwanted emotions, combined with a simultaneous difficulty in identifying and contextualizing these emotions. In addition, dysfunctional strategies of emotion regulation, i.e., substance abuse or suicidal self-directed violence, are used [2,6,7]. The goal of therapeutic interventions for both SUD and BPD could, therefore, be to learn that activated unwanted emotions can be regulated and positive emotions can be strengthened [6,8]. Success here depends largely on one’s own mindfulness and is rewarded by positive emotions and the experience of self-efficacy in regulating one’s own emotions [2,4].

Animal-assisted interventions can be applied to achieve the goals described above. By definition, animal-assisted treatment, animal-assisted education, and animal-assisted support programs are categorized under the term animal-assisted services [9]. They are defined as services provided by a health or social service provider or educator working with a specially trained animal to offer therapeutic, educational, and/or supportive processes with the aim of improving people’s well-being. The welfare of the animal is also ensured. In studies in animal-assisted services, there are often methodological shortcomings and a lack of consistent terminology [9,10]. Shortcomings, such as small sample sizes, lack of randomization, standardization, and manualization of interventions, and the use of non-specific outcome measures, are reported [10,11,12]. Nevertheless, both reviews and clinical studies on animal-assisted treatment (AAT) in people with mental disorders indicate significant benefits, such as improvements in the areas of emotionality (positive and negative emotions), pathologies (anxiety, depression, and pain), social behavior, level of functioning, mindfulness, and self-efficacy expectancy [11,12,13,14,15,16,17,18,19,20,21,22].

Based on the existing literature, the following question arises: which animals and contexts are suitable for achieving the above-mentioned goals? Previous studies have achieved good results in AAT with sheep in terms of reducing negative emotions [14,15], improving overall quality of life [15], and improving positive emotions, mindfulness, and self-efficacy expectancy [14]. Sheep are ideal for inclusion in AAT due to their gentle, quiet nature, social structure, and sensitivity [23]. They have social behavioral skills and are herd animals with high vigilance due to the risk of predation, but humans can be integrated into their social structure. They are able to communicate their moods and willingness to interact [23]. Due to these aspects, high mindfulness skills are required when interacting with sheep [15,24], and, due to their willingness to engage in social interactions, an emotional bond can develop. This creates an atmosphere of safety and relaxation in the human–animal interaction for both the sheep and the humans. Within AAT practice, there is a growing awareness that the health and well-being of animals should also be a central focus. One approach to this is the One Health Framework, which includes the welfare of animals, humans, and the environment at a high standard [25].

Only a few studies have been published involving sheep in AAT. Sheep have been part of AAT involving farm animals [26,27,28], in a sheep rearing unit [29], in a pilot study as part of multi-session AAT [30], and also in a multi-session AAT study with one-year follow-up [15]. The results were all promising. Patients with various mental disorders showed improvements in the above-mentioned outcomes after the intervention.

In an initial study of our own with sheep in AAT [14], we were able to demonstrate that a single AAT session with sheep in a group setting could activate positive emotions and even reduce negative emotions in psychiatric inpatients with emotion dysregulation, i.e., SUD. It was also observed that mindfulness and self-efficacy expectancy could be significantly increased through AAT with sheep. The aims of the present study were as follows: 1. Replication (to examine whether the results of our initial AAT study in participants with SUD could be replicated), 2. applicability to participants with BPD (to examine another group of inpatients with maladaptive emotion regulation, namely BPD), and 3. comparison of SUD and BPD (to examine possible difference in the effectiveness of AAT depending on the type of disorder (SUD vs. BPD)).

## 2. Materials and Methods

### 2.1. Study Design

We conducted a randomized controlled trial with repeated measures, comparing treatment as usual (TAU) with a manualized animal-assisted treatment in addition to TAU (TAU+AAT) in two different groups of psychiatric inpatients with emotion dysregulation, one with SUD and one with BPD.

Data were collected before the intervention (PRE) and directly after the intervention (POST). Two follow-ups, one week later and one month later, were also conducted. As this data is not complete, it will be published later. Ethical approval was obtained from the ethics committee of the University of Ulm (no. 45/23). In accordance with the Declaration of Helsinki, participants were informed and gave their written informed consent. The study was registered in the German Registry for Clinical Studies (DRKS00031347, date of first registration 20 April 2023).

### 2.2. Participants

The participants were psychiatric inpatients with a disorder in emotion regulation and were from two different departments of a university hospital for psychiatry and psychotherapy in Germany. Ward one specializes in treating people with BPD who require CBT-based inpatient psychotherapy due to an acute mental health crisis, including aggressive behavior (self-harm or harm to others). Ward two specializes in treating patients with SUD and comorbid disorders. These patients with SUD have already completed withdrawal treatment and are now receiving further CBT-based psychotherapy.

**Figure 1:**
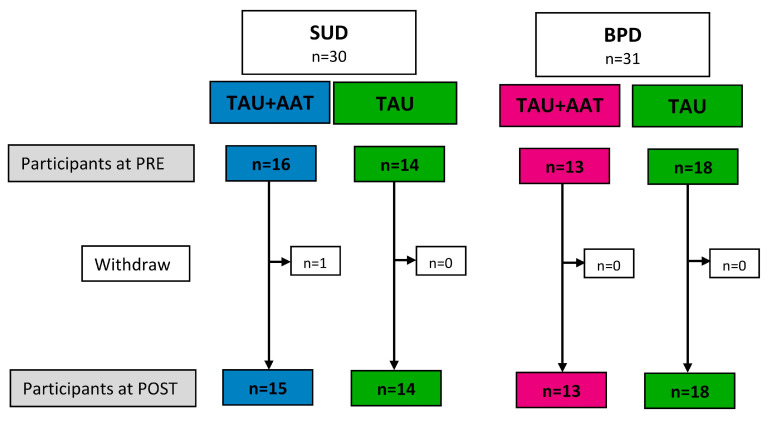
CONSORT for the recruitment of the two groups, animal-assisted treatment (TAU+AAT) and treatment as usual (TAU), separated for participants with substance use disorder (SUD) and participants with borderline personality disorder (BPD).

Inclusion criteria were an age between 18 and 65 years, continuing inpatient treatment for at least another 7 days, the ability to give informed study consent, and physical requirements, such as being able to stand and walk safely. Exclusion criteria were allergies, animal phobia, and aversion to specific animals [21]. Also excluded were inpatients with acute psychoses or suicidality, as these patients were not sufficiently resilient. After giving informed consent, participants in both groups (SUD and BPD) were randomly assigned to the TAU+AAT or TAU group (see Figure 1). One participant withdrew due to an acute crisis.

### 2.3. The General Framework of the AAT Procedure

The One Health Framework is an approach that considers the health of humans, animals, and the environment [25]. Our study was based on quality standards for the inclusion of animals in animal-assisted services [31,32,33,34] and on an adapted risk assessment tool for animal–human interaction [35].

The AAT took place at a farm specializing in AAT with sheep in southern Germany named “Prinzenhof” and was conducted by three certified specialists. PP is a specialist in animal-assisted education and support. She is the owner of the sheep, was primarily responsible for the welfare and supervision of the sheep during the AAT, and looked out for signs of stress in the sheep. CN is a specialist in AAT and a licensed nurse. During the AAT, she was primarily responsible for conducting the intervention. This duo was accompanied by an additional licensed therapist from the ward where the respective study participants were currently being treated. Accordingly, this person was responsible for the study participants and for transferring their experiences into the psychotherapeutic process during their hospital stay.

The sheep involved in this study were raised at the “Prinzenhof” and live there as a herd. The care and welfare of the animals are of high priority, according to the standards of animal welfare [23,31]. Sheep are normally flight-prone animals, but the sheep at the “Prinzenhof” are accustomed to human interaction since birth and have been trained for AAT by PP. The breeds of the four sheep were Mountain Sheep mix, Mountain Sheep–Valais mix, Moorland Sheep, or Coburg Fox Sheep mix. The age of the sheep was between 3 and 5 years. Further details on the sheep are described in Table 1.

### 2.4. The Sections of the AAT Procedure

The AAT procedure consisted of a single AAT session in a group and lasted approximately 5 h, including travel time, summing 1.5 h. Before the AAT began on the farm, the four participants introduced themselves to each other, which also provided an opportunity to address any fears or concerns. The aim was to promote group cohesion and thus create an optimal learning environment. The entire group thus consisted of four participants, four sheep, and three AAT specialists. In good weather conditions, the AAT took place in the sheep paddock; in bad weather conditions, it took place in the open stable. The AAT procedure was manualized and divided into seven sections:

- *Observation of PP’s interaction with the sheep (approx. 15 min):* The participants observed PP interacting with the sheep in the separated sheep paddock. This interaction served as a model for respectful treatment of the sheep’s basic needs and for mindfulness.
- Introduction of the sheep (approx. 30 min): The sheep, still separated by a fence, were introduced individually by name and specific characteristics, which made it easier for participants to connect with them.
- *Approach via feeding (approx. 15 min):* The participants fed the sheep over the fence, establishing initial physical contact.
- *Approach via presence (approx. 30 min)*: The participants sat on prepared tree trunks in the paddock. Contact was initiated exclusively by the freely moving sheep. When a sheep approached a participant, the participant was allowed to touch and pet the sheep. The decision on the duration of the contact was left to the sheep. In most cases, each partnership between sheep and human was established here for the entire session.
- *Experience of competence and attachment (approx. 15 min):* In order to walk 200 m together as a sheep–participant duo, the participants had the task of putting the sheep on a leash.
- *Free walk in mindful interaction (approx. 45 min):* Afterwards, the leash was removed, and all sheep walked the rest of the distance together with all the participants. The sheep and participants formed a common flock, which made it possible to experience the connection between the sheep and humans. The participants had the opportunity to interact in a mindful manner, individually with the sheep, and experience the trust that had been built between them.
- *Farewell (approx. 30 min):* The sheep were taken back to the paddock, given water, and bid farewell by the participants. The participants conducted a feedback session over snacks and drinks.

### 2.5. Materials

After positive and negative emotional states, mindfulness, and self-efficacy expectancy had been identified as variables that could be targeted in AAT with sheep, these variables were assessed as outcomes. For the primary outcome, the State–Trait Anxiety Inventory, state version (STAI-S [36,37]), was used to measure positive and negative emotions. It consists of 20 four-point Likert-scaled items. A total score for positive emotions and a total score for negative emotions were calculated.

For secondary outcomes, the Freiburg Mindfulness Inventory short version (FMI) and the General Self-Efficacy Short Scale (ASKU) were used. The FMI [38,39] was applied to assess mindfulness. The FMI consists of 14 four-point Likert-scaled items resulting in a sum score. For measuring self-efficacy expectancy, the ASKU [40] was used. It consists of 3 items with ratings from 1 to 5. A mean value was calculated for the analysis. All outcomes were collected at PRE and POST.

The Objective Social Outcome Index (SIX [41]) was used to assess social integration at PRE. The SIX consists of 4 items: employment, accommodation, partnership/family, and friendship. The resulting sum score ranged from 0 to 6. Data on socio-demographic variables (age, sex) and clinical variables (diagnoses, duration of inpatient stay) were extracted from the medical records after discharge.

### 2.6. Power Calculation

Based on the results of the previous study [14], an effect size of Cohen’s d = 1.0 was calculated for the primary outcome STAI-S positive emotions sum score. G*Power^®^ (version 3.1) was used to calculate the required sample size, with 1-ß = 0.8 for one-sided testing with alpha = 0.05. For 2 groups (TAU+AAT vs. TAU resp. SUD vs. BPD) and using 2 measurement times (PRE, POST), a sample size of n = 14 per group when running *t*-tests for independent groups was required.

### 2.7. Statistical Analyses

The analyses were performed using IBM SPSS 29^®^. First, the comparability of the corresponding subsamples was examined. Dichotomous variables were evaluated with the Chi^2^-test. Normal distribution was tested with the Kolmogorov–Smirnov test. Group differences were analyzed for normally distributed data with *t*-tests for independent groups. Variance homogeneity was analyzed with Levene’s test. For non-normally distributed data, group differences were tested by the Mann–Whitney U test. The two TAU+AAT groups for SUD and BPD differed in terms of sex and number of additional diagnoses. These two variables were therefore included as covariates in further comparative analyses.

To examine the research questions, repeated-measures ANOVAs were calculated for the outcome sum scores of the STAI-S (negative and positive emotions), the FMI sum score, and the ASKU mean value. As Field [42] indicated, ANOVA is considered a robust procedure even when the assumption of normal distribution is violated and could, therefore, be used. The assumption of sphericity was tested using Mauchly’s test of Sphericity and was not violated. Cohen’s d was calculated for the effect size, where d = 0.2–0.5 represents a small effect size, d = 0.5–0.8 represents a moderate effect size, and d > 0.8 represents a large effect [43]. To examine the effectiveness of the TAU+AAT, Wilcoxon tests were calculated post hoc, separately for participants with SUD and BPD. The correlation coefficient r was calculated for the effect size, whereby according to Cohen [43], r = 0.1 is regarded as a small effect, r = 0.3 as a medium effect, and r = 0.5 as a large effect.

## 3. Results

### 3.1. Question 1: Replication for Participants with SUD

To replicate the study by Schmid et al. [14], the comparability of the TAU+AAT and TAU groups was first tested after randomization. The n = 29 participants with SUDs in the two groups did not differ in any of the demographic and clinical data (see Table 2, SUD section).

**Figure 2:**
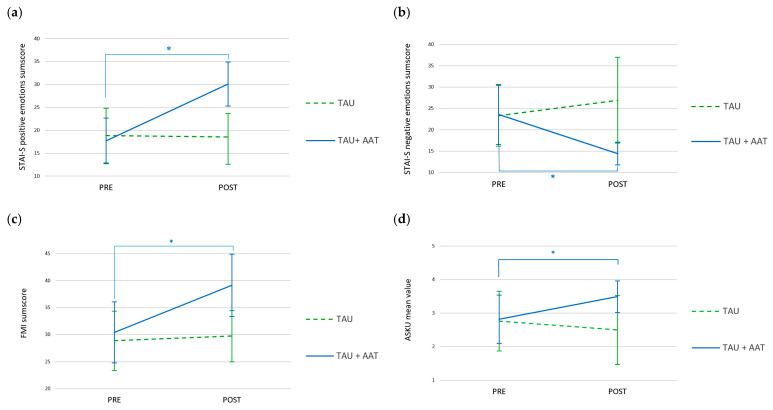
Values of positive (**a**) and negative (**b**) emotions sum scores (STAI-S), as well as mindfulness sum score (**c**) and self-efficacy expectancy mean value (**d**) over time (PRE vs. POST intervention) for both groups (TAU+AAT vs. TAU) among participants with SUD. *: significant time × group interaction and significant time effect in TAU+AAT in all four outcomes.

In the primary outcome, the STAI-S positive emotions sum score, there was a significant interaction between time and group with a large effect (F(1,27) = 52.630; *p* < 0.001; Cohen’s d = 1.396). This was also observed in the STAI-S negative emotions sum score (F(1,27) = 18.236; *p* < 0.001; Cohen’s d = 0.822). Over time, the STAI-S positive emotions sum score increased significantly in the TAU+AAT group, in contrast to the TAU group. In the STAI-S negative emotions sum score, the mean of the TAU+AAT group decreased significantly over time, which was not observed in the TAU group (see Figure 2a,b). The two secondary outcomes, the mindfulness sum score and self-efficacy expectancy mean value, also displayed significant time × group interactions (mindfulness: (F(1,27) = 14.470; *p* < 0.001; Cohen’s d = 0.550; self-efficacy expectancy: F(1,27) = 14.266; *p* < 0.001; Cohen’s d = 0.555), both with medium effect sizes. Here, again, both groups started at a comparable level before the intervention. Over time, the TAU+AAT group increased significantly, while the TAU group did not improve over time in mindfulness and self-efficacy expectancy (Figure 2c,d).

Post hoc analyses demonstrated that participants in the TAU+AAT group improved significantly over time (PRE vs. POST) in all four outcomes, each with a large effect (STAI-S positive sum score: *p* < 0.001; r = 0.880; STAI-S negative sum score: *p* < 0.001; r = 0.880; FMI sum score: *p* < 0.01; r = 0.815; ASKU mean value: *p* < 0.001; r = 0.712).

### 3.2. Question 2: Applicability to Participants with BPD

To investigate whether the results of the participants with SUD could also be achieved in the participants with BPD, the TAU+AAT and TAU groups in the participants with BPD were examined. There were no differences in the demographic and clinical data for the n = 31 participants with BPD in the TAU+AAT group compared to the TAU group (see Table 2, BPD section).

**Figure 3:**
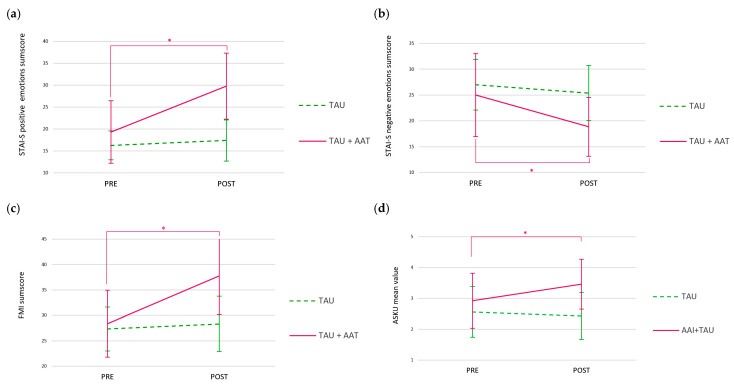
Values of positive (**a**) and negative (**b**) emotions sum scores (STAI-S), as well as mindfulness sum score (**c**) and self-efficacy expectancy mean value (**d**) over time (PRE vs. POST intervention) for both groups (TAU+AAT vs. TAU) among participants with BPD. *: significant time × group interaction and significant time effect in TAU+AAT in all four outcomes.

In the primary outcome, participants with BPD also demonstrated significant interaction effects between the groups (TAU+AAT vs. TAU and time (PRE vs. POST). The TAU+AAT group achieved a significantly greater increase in the STAI-S positive emotions sum score compared to the TAU group (F(1,29) = 28.522; *p* < 0.001; Cohen’s d = 0.992), with a large effect (see Figure 3a). In the STAI-S negative emotions sum score, the TAU+AAT group also reported a significantly greater reduction over time than the TAU group (F(1,29) = 15.742; *p* < 0.001; Cohen’s d = 0.4312) (see Figure 3b). In the secondary outcomes, the mindfulness score (FMI) and the self-efficacy expectancy mean value (AKSU), the TAU+AAT group improved significantly more over time than the TAU group (FMI sum score: F(1,29) = 31.466; *p* < 0.001; Cohen’s d = 1.041; AKSU mean value: F(1,29) = 6.798; *p* < 0.05; Cohen’s d = 0.484) (see Figure 3c,d).

In post hoc analyses, the participants with BPD improved significantly over time in all four outcomes (PRE vs. POST), with large effects (STAI-S positive sum score: *p* < 0.01; r = 0.852; STAI-S negative sum score: *p* < 0.01; r = 0.806; FMI sum score: *p* < 0.001; r = 0.883; ASKU mean value: *p* < 0.05; r = 0.648).

### 3.3. Question 3: Comparison of Participants with TAU+AAT in the Two Groups SUD vs. BPD 

To investigate the third question, only the TAU+AAT participants in the two groups (SUD vs. BPD) were compared (see Table 3). The two groups (SUD vs. BPD) differed significantly in terms of sex (Chi^2^ (1,28) = 8.299; *p* < 0.01) and in the number of additional diagnoses (t(17.857) = 7.589; *p* < 0.001), so these two variables were treated as covariates in the further analyses.

**Figure 4:**
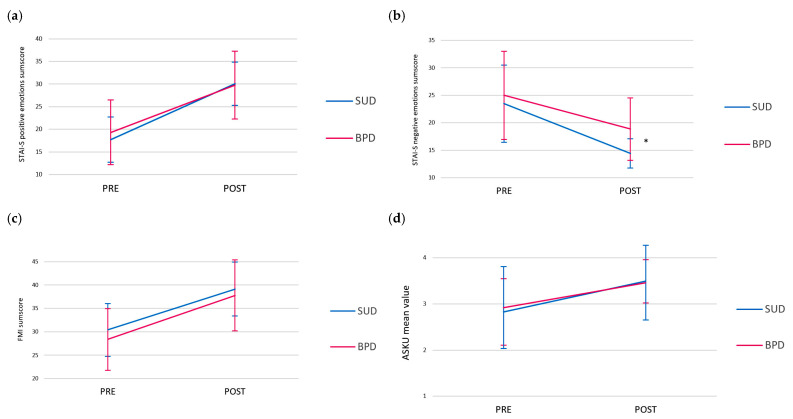
Values of positive (**a**) and negative (**b**) emotions sum scores (STAI-S), as well as mindfulness sum score (**c**) and self-efficacy expectancy mean value (**d**) over time (PRE vs. POST intervention) for both TAU+AAT groups (SUD vs. BPD). *: significant group effect in STAI-S negative emotions sum score.

For all outcomes, only the STAI-S negative sum score demonstrated a significant group effect (F(1,24) = 5.748; *p* < 0.05; Cohen’s d = 0.488). The participants with BPD had significantly higher scores at PRE and POST compared to participants with SUD. With the exception of this effect, no significant differences were found between the participants in the two groups (SUD vs. BPD) (see also Figure 4a–d).

## 4. Discussion

In a randomized controlled trial, we first sought to replicate the results of our initial pilot study [14], in which a single session of AAT with sheep significantly improved positive emotions and reduced negative emotions in inpatients with SUD. Secondly, we aimed to investigate the study’s applicability in participants with BPD. Thirdly, we were interested in determining whether there is a difference in the effectiveness of AAT depending on the type of disorder (SUD vs. BPD).

The significant results of our initial study with participants with SUD were replicated in the current study. Significant effects between PRE and POST, with large effect sizes in all four outcomes (positive and negative emotions, mindfulness, and self-efficacy expectancy) were confirmed. This is consistent with findings in previous AAT studies on emotionality [13,14,15,18,20,21,22], mindfulness [14,30], and self-efficacy [14,26]. Remarkably, all studies except our own initial study [14] used a more extensive AAT design (with 9 sessions [15,30], 24 sessions [26], and 34 sessions [28]) to achieve similar effects. In our initial, as well as in our current study, the results were achieved using a single-session AAT procedure.

In the current study, we decided to expand the investigation to another group of inpatients. In the literature, good results were reported for psychiatric patients in general [22,26], but also specifically for patients with depression [13,15,21,30], PTSD [16], schizophrenia [20], or SUD [18]. One of the therapeutic effects of AAT is to promote positive emotions and regulate negative emotions in a mindful way, which can prevent dysfunctional behavior, such as substance use or self-injury. AAT is, therefore, suitable for the treatment of participants with difficulties in emotion regulation. Since these issues represent problem areas in the treatment of SUD and BPD [6,8], we expanded the sample to include participants with BPD. As expected, large effect sizes were also found here in the reduction of negative emotions and the improvement of positive emotions, mindfulness, and self-efficacy. In a direct comparison of the two disorders, AAT yielded a very similar pattern of outcomes, although participants with BPD displayed higher negative emotion scores compared to participants with SUD. As already mentioned, both disorders are characterized by impaired emotion regulation. Accordingly, psychotherapeutic goals should focus on teaching skills for successful up- and down-regulation (to prevent dysfunctional behavior) and mindfulness skills (to avoid becoming overwhelmed) [4]. As has been demonstrated repeatedly, an enhanced psychotherapeutic intervention with AAT can apparently support this process.

As mentioned earlier, sheep are normally flight-prone animals with high vigilance due to the risk of predation. Working with sheep, therefore, requires a high degree of mindfulness skills to ensure successful human–animal interaction [15,24,44]. Our AAT procedure is tailored to this characteristic. It is undisputed that other animals are also suitable for inclusion in AAT [10,11,16,19]. The key to successful AAT seems to be, above all, a good fit between the animal species, the characteristics of the animal, the treatment goals, the AAT procedure, the participant sample, and the therapists.

In the future, it also seems essential to take animal welfare into account, more specifically in line with the One Health Framework. There are initial promising methodological approaches for measuring the stress that animals experience during AAT [45].

This is, to our knowledge, the first study that replicated in an RCT the results of AAT with sheep in psychiatric inpatients. Since animal-assisted services studies in general often do not meet high methodological standards (see also [14]), not only good methodological studies but also their replications are important. However, our study has several limitations. As described above, the fit between species, animals, treatment goal, procedure, sample, and therapist is an important factor for successful AAT. However, this noticeably limits the generalizability of our results. In addition, the sample size in the current study is small. The main limitation is certainly the question of the sustainability of the effects achieved. Even if there are significant changes in the variables surveyed immediately after the AAT, it remains unclear whether these effects are maintained over time. Unfortunately, the follow-up data for the current study is not yet complete, which is why no statements can be made yet.

## 5. Conclusions

AAT, administered as described, appears to be effective in up- and down-regulating emotions, as well as in improving mindfulness and self-efficacy expectancy in psychiatric inpatients with difficulties in emotion regulation, at least for a short period of time. Furthermore, sheep seem to be suitable for AAT with a focus on mindfulness. In the treatment of people with difficulties in emotion regulation, mindfulness-based approaches could be a therapeutic extension.

## Figures and Tables

**Table 1:** Description of the sheep.

Name	Breed, Year of Birth, Sex	Characteristics/Personality
Zenzi (Figure S1)	Mountain Sheep–Valais mix, 2019, ewe	Very sensitive, curious, and human oriented; conveys great calmness and appreciation
Anni (Figure S2)	Mountain Sheep, 2019, ewe	Athletic, curious, bright, gentle, and confident
Toni (Figure S3)	Coburg Fox Sheep mix, 2020, ewe	Observant, self-confident, strong-minded, and quick learner
Sissi (Figure S4)	Moorland Sheep, 2020, ewe	Sensitive, sets clear boundaries, and likes to be pampered

**Table 2:** Group comparison of participants receiving animal-assisted treatment (TAU+AAT) versus participants receiving treatment as usual (TAU), separated into participants with substance use disorder (SUD) and participants with borderline personality disorder (BPD).

			TAU+AAT	TAU	*p*
SUD	*n*		15	14	
	age	M (SD)	37.67 (14.04)	40.07 (12.88)	0.561 ^1^
	female	*n* (%)	6 (40.0%)	7 (50.0%)	0.715 ^2^
	number of additional diagnoses	M (SD)	7.73 (3.11)	6.00 (2.57)	0.093 ^1^
	duration of stay (in days)	M (SD)	44.33 (26.19)	42.00 (14.64)	0.747 ^1^
	social integration (SIX)	M (SD)	3.27 (1.34)	3.00 (1.52)	0.715 ^1^
BPD	*n*		13	18	
	age	M (SD)	33.69 (11.91)	29.44 (9.76)	0.417 ^1^
	female	*n* (%)	12 (92.3%)	18 (100.00%)	0.419 ^2^
	number of additional diagnoses	M (SD)	1.23 (1.09)	1.44 (1.15)	0.650 ^1^
	duration of stay (in days)	M (SD)	77.08 (121.93)	42.06 (34.00)	0.540 ^1^
	social integration (SIX)	M (SD)	3.69 (1.44)	3.61 (1.85)	0.953 ^1^

Notes. SIX: Objective Social Outcome Index; ^1^: Mann–Whitney U test; ^2^: Chi^2^-test.

**Table 3:** Group comparison of participants with animal-assisted treatment (TAU+AAT) between participants with substance use disorder (SUD) and borderline personality disorder (BPD).

		SUD	BPD	*p*
*n*		15	13	
age	M (SD)	33.67 (14.04)	33.69 (11.91)	0.431 ^1^
female	*n* (%)	6 (40.0%)	12 (92.3%)	0.006 ^2^
number of secondary diagnoses	M (SD)	7.73 (3.11)	1.23 (1.09)	0.001 ^1^
duration of stay (in days)	M (SD)	44.33 (26.19)	77.08 (121.93)	0.928 ^3^
social integration (SIX)	M (SD)	3.27 (1.34)	3.69 (1.44)	0.496 ^3^

Notes. SIX: Objective Social Outcome Index; ^1^: *t*-test for independent ^2^: Chi^2^-test; ^3^: Mann–Whitney U test.

## Data Availability

The data presented in this study are available on request from the corresponding author, as the study is part of an ongoing research project.

## Supplementary Materials

The following supporting information can be downloaded at: https://www.mdpi.com/article/10.3390/healthcare13212808/s1, Figure S1: Zenzi; Figure S2: Anni; Figure S3: Toni; Figure S4: Sissi.
