# Supplementary figures and images for: “The Sheep Did It Again”: Replication of Animal-Assisted Treatment in Psychiatric Inpatients with Substance Use Disorder and Borderline Personality Disorder in a Randomized Controlled Trial
Source: Healthcare (Basel). 2025 Nov 5;13(21):2808. doi: 10.3390/healthcare13212808 (PMC12609190; doi:10.3390/healthcare13212808)

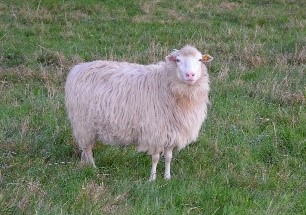

Supplement: Supplementary file 1 [file healthcare-13-02808-s001.zip › Sissi.jpeg]

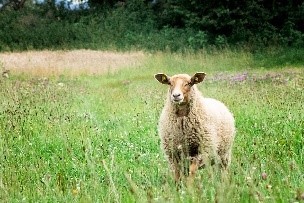

Supplement: Supplementary file 1 [file healthcare-13-02808-s001.zip › Toni.jpeg]

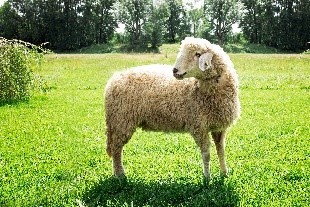

Supplement: Supplementary file 1 [file healthcare-13-02808-s001.zip › Zenzi.jpeg]

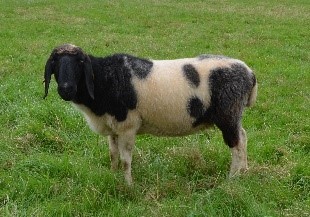

Supplement: Supplementary file 1 [file healthcare-13-02808-s001.zip › Anni.jpeg]
